# Indole Propionic Acid, an Unusual Antibiotic Produced by the Gut Microbiota, With Anti-inflammatory and Antioxidant Properties

**DOI:** 10.3389/fmicb.2020.575586

**Published:** 2020-10-27

**Authors:** Dereje Abate Negatu, Martin Gengenbacher, Véronique Dartois, Thomas Dick

**Affiliations:** ^1^Center for Innovative Drug Development and Therapeutic Trials for Africa, College of Health Sciences, Addis Ababa University, Addis Ababa, Ethiopia; ^2^Center for Discovery and Innovation, Hackensack Meridian Health, Nutley, NJ, United States; ^3^Department of Medical Sciences, Hackensack Meridian School of Medicine, Nutley, NJ, United States; ^4^Department of Microbiology and Immunology, Georgetown University, Washington, DC, United States

**Keywords:** antibiotic, gut microbiota, *Mycobacterium*, tuberculosis, non-tuberculous mycobacteria, host-directed therapy

## Abstract

Most antibiotics are produced by soil microbes and typically interfere with macromolecular synthesis processes as their antibacterial mechanism of action. These natural products are often large and suffer from poor chemical tractability. Here, we discuss discovery, mechanism of action, and the therapeutic potentials of an unusual antibiotic, indole propionic acid (IPA). IPA is produced by the human gut microbiota. The molecule is small, chemically tractable, and targets amino acid biosynthesis. IPA is active against a broad spectrum of mycobacteria, including drug resistant *Mycobacterium tuberculosis* and non-tuberculous mycobacteria (NTM). Interestingly, the microbiota-produced metabolite is detectable in the serum of healthy individuals, tuberculosis (TB) patients, and several animal models. Thus, the microbiota in our gut may influence susceptibility to mycobacterial diseases. If a gut-lung microbiome axis can be demonstrated, IPA may have potential as a biomarker of disease progression, and development of microbiota-based therapies could be explored. In addition to its antimycobacterial activity, the molecule displays anti-inflammatory and antioxidant properties. This raises the possibility that IPA has therapeutic potential as both antibiotic and add-on host-directed drug for the treatment of TB in patient populations where disease morbidity and mortality is driven by excessive inflammation and tissue damage, such as TB-associated immune reconstitution inflammatory syndrome, TB-meningitis, and TB-diabetes.

## Introduction

To identify chemical starting points for the discovery of new drugs against resistant tuberculosis (TB) and lung disease caused by non-tuberculous mycobacteria (NTM), we recently screened a library of rule-of-3 (R03) compliant compounds for whole cell actives (Negatu et al., 2018). R03 compliant compounds are “fragment”-sized (i.e., molecular weight <300 g/mol), have a cLogP of ≤3, and the number of hydrogen bond donors and acceptors is ≤3 (Jhoti et al., 2013). Due to their small size and the resulting limited molecular interaction surface, R03 compliant compounds are expected to show poor on-target activity. However, R03 compounds constitute attractive starting points for lead optimization (Jhoti et al., 2013; Gopal and Dick, 2014). Typically, R03 libraries are employed for structure-based drug discovery approaches in which hits are either grown or connected to generate high affinity binders (Jhoti et al., 2013). It is interesting to note that several anti-TB drugs, such as pyrazinamide and isoniazid, are fragment-sized drugs (Jhoti et al., 2013; Riccardi and Pasca, 2014). The physicochemical properties associated with their size makes these small drugs effective penetrators of lung lesions associated with mycobacterial diseases. Thus, fragment-sized TB drugs effectively reach all mycobacterial populations sequestered in various lesion compartments (Prideaux et al., 2015). This lesion-pharmacokinetic property contributes to the remarkable clinical efficacy of pyrazinamide, despite the drug’s moderate *in vitro* potency (Dartois, 2014; Prideaux et al., 2015).

Screening the R03 library resulted in the identification of indole propionic acid (IPA) as a new antimycobacterial (Negatu et al., 2018). The compound showed anti-TB (including multi-drug resistant *Mycobacterium tuberculosis*) and anti-NTM (including *Mycobacterium avium*) activity *in vitro*. Using a mouse model of TB infection, we demonstrated *in vivo* efficacy (Negatu et al., 2018). IPA showed no activity against Gram-negative or -positive bacteria, and thus appears to display selective but broad spectrum antimycobacterial activity (Negatu et al., 2018, 2019).

Interestingly, IPA is a metabolite produced by gut bacteria (Dodd et al., 2017). Recently, several IPA producing gut-dwelling clostridia and *Peptostreptococcus anaerobius* were identified, and the biosynthetic pathway of IPA production from tryptophan was elucidated (Dodd et al., 2017). Thus, IPA is a microbial metabolite that inhibits growth of other microbes and therefore represents an antibiotic in the classical sense (Negatu et al., 2018). Our clinical antibacterial arsenal is composed to a large extent of natural products or their semi-synthetic derivatives. These antibiotics are mostly derived from soil bacteria, often *Streptomyces* (Clardy et al., 2009; Genilloud, 2017). They are usually large and of complex chemistry, with the associated pharmacokinetic and synthetic chemistry issues, presenting many challenges for medicinal chemists (Leeson and Springthorpe, 2007). IPA is an unusual antibiotic as it is produced by gut bacteria rather than soil bacteria. Furthermore, IPA is small, and the indole-based scaffold is chemically tractable.

## IPA Blocks Mycobacterial Tryptophan Biosynthesis by Mimicking Tryptophan as Allosteric Inhibitor

**Figure 1:**
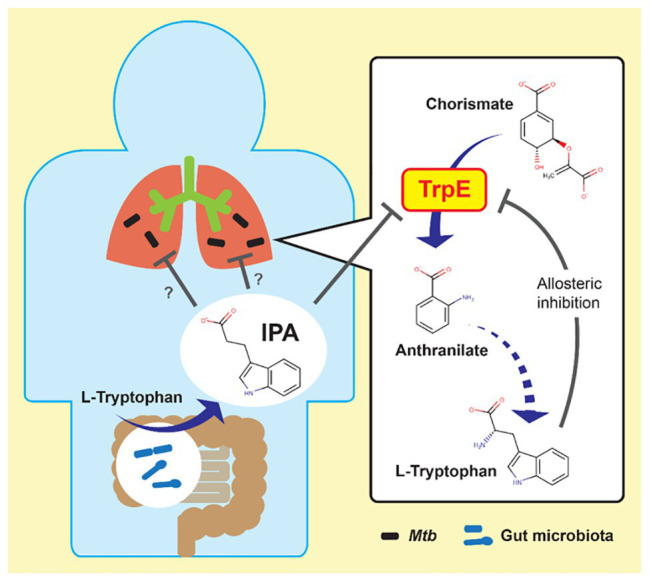
Possible link between gut microbiota and mycobacterial lung disease. Left: indole propionic acid (IPA) is produced by gut bacteria from tryptophan and enters the blood stream. Right: IPA inhibits mycobacterial tryptophan biosynthesis by targeting anthranilate synthase TrpE, mimicking tryptophan as allosteric inhibitor. Mtb, *Mycobacterium tuberculosis*.

*In vitro* potency and *in vivo* efficacy of IPA are moderate (Negatu et al., 2018). Its mechanism of action was determined by our group to enable rational, target-based optimization. IPA is a deamination analog of tryptophan, and thus structurally similar to this aromatic amino acid. Tryptophan biosynthesis is an essential pathway in mycobacteria *in vitro* as well as *in vivo* (Zhang et al., 2013; Wellington et al., 2017). The pathway is regulated *via* a negative feedback loop in which the end product tryptophan acts as allosteric inhibitor of the first committed enzymatic step of the pathway catalyzed by anthranilate synthase TrpE (Bashiri et al., 2015). Thus, we hypothesized that IPA may mimic tryptophan as allosteric inhibitor and block tryptophan synthesis. Through structural modeling followed by metabolic, genetic, and biochemical analyses, we demonstrated that IPA indeed blocks tryptophan biosynthesis by binding to the allosteric tryptophan binding site of TrpE, shutting down the enzyme’s activity (Figure 1; Negatu et al., 2019). Thus, IPA acts by decoupling a bacterial regulatory feedback mechanism. The antibiotic mimics tryptophan as allosteric inhibitor, thereby switching off production of this aromatic amino acid regardless of intracellular tryptophan levels. The identification of the molecular target of IPA provides the platform for rational chemical optimization in which semisynthetic analogs with increased potency can be designed to develop a new class of broad-spectrum antimycobacterials.

Blocking an amino acid biosynthetic pathway is a novel antibiotic mechanism. Most antibiotics, including synthetic antibacterials, target macromolecular synthesis processes such as protein (e.g., streptomycin), RNA (e.g., rifamycins), or peptidoglycan synthesis (e.g., beta-lactams; Clardy et al., 2009). Only few synthetic antibacterials interfere with bacterial metabolism. A classic example is trimethoprim, a dihydrofolate reductase inhibitor, blocking folate biosynthesis (Hitchings, 1973). A recent example is pyrazinamide, an aspartate decarboxylase degrader, blocking coenzyme A biosynthesis in *M. tuberculosis* (Gopal et al., 2017, 2020).

## IPA, A Link between Gut Microbiota and Mycobacterial Lung Disease?

IPA is well-known as a metabolite produced by human gut bacteria (Young et al., 1980). It can be detected systemically in blood, and thus reaches all major organs after migrating from the gut to the bloodstream (Young et al., 1980; Wikoff et al., 2009). Recent observations suggest that the gut microbiota may affect TB progression by modulating the host immune response (Wood et al., 2017; Naidoo et al., 2019). The discovery of IPA’s antimycobacterial activity may provide the missing functional link between gut bacteria and mycobacterial lung disease (Figure 1). Analyses of serum of healthy volunteers show large (up to 1,000-fold) person-to-person variability (Wikoff et al., 2009; unpublished observations of the authors). IPA can be detected in the serum of animal models, which are used to study mycobacterial lung disease, such as mice and rabbits (Wikoff et al., 2009; Kennedy et al., 2018; unpublished observations by the authors). Hence, we have the models available to study a possible IPA-related microbiota effect on mycobacterial lung diseases. Furthermore, studies can be carried out with human cohorts to determine whether there is a correlation between disease susceptibility or disease progression and IPA levels in the serum. If these studies do show correlations, IPA may be useful as biomarker (Naidoo et al., 2019).

These studies may open avenues for the development of microbiota-based therapies (Reardon, 2014; Mimee et al., 2016; Suez and Elinav, 2017) using IPA producing strains as probiotics, possibly supplemented with high content tryptophan (the substrate for IPA production; Dodd et al., 2017) foods. In addition to using natural IPA producers, generation of recombinant probiotics could be considered by incorporating the gene cluster that converts tryptophan to IPA (Mathipa and Thantsha, 2017). Increased levels of endogenously produced IPA may complement standard, exogenously supplied antimycobacterials.

In addition to targeting the pathogen directly *via* inhibiting tryptophan biosynthesis, increased IPA levels may also act indirectly on the disease *via* host directed mechanism, as discussed in the next sections.

## IPA, Utility as Host-Directed Therapy?

**Figure 2:**
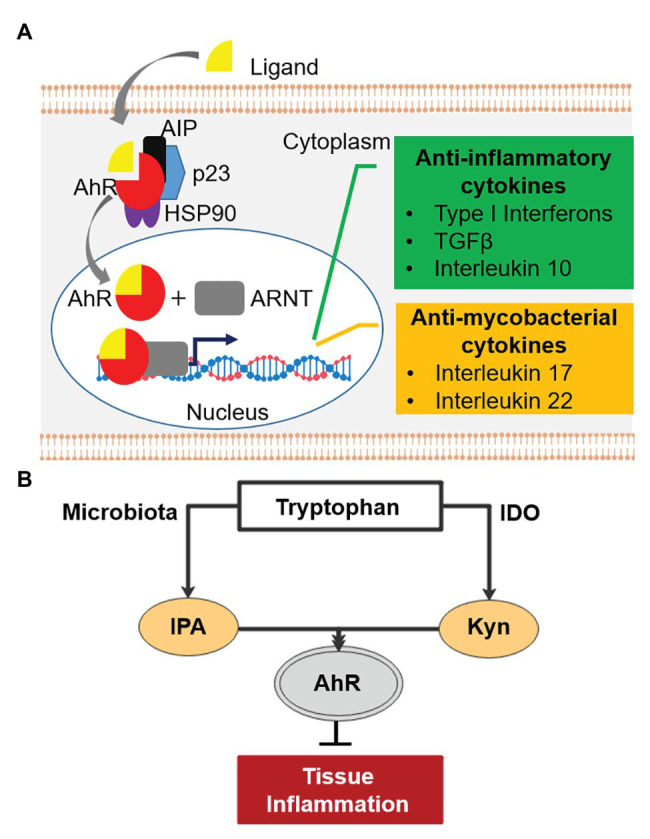
Aryl hydrocarbon receptor (AhR) signaling pathway and tryptophan-indoleamine 2,3-dioxygenase (IDO)-AhR axis. **(A)** AhR (red) is localized in the cytosol of human cells in complex with other proteins. Upon activation by ligands (e.g., tryptophan metabolites), AhR is released from the complex and binds its partner, the AhR nuclear translocator (ARNT) in the nucleus. The complex of AhR and ARNT binds upstream of target genes and enhances the expression of anti-inflammatory (green) and “antimycobacterial” cytokines (orange). AIP, AhR-interacting protein; p23, chaperone; HSP90, 90kDa heat shock protein. **(B)** Tryptophan-IDO-AhR axis in inflammation.

Before the discovery of IPA’s antibiotic activity, the molecule and other tryptophan metabolites were shown to have immune modulatory properties (Gao et al., 2018; Nicolas and Chang, 2019). Tryptophan metabolites are potent inducers of the Aryl hydrocarbon Receptor (AhR), a central player in the regulation of both innate and adaptive immune responses (Stockinger et al., 2014; Gao et al., 2018; Gutierrez-Vazquez and Quintana, 2018; Nicolas and Chang, 2019). AhR is a ligand-activated transcription factor localized in the cytoplasm of human cells in complex with other proteins (Figure 2A; Gutierrez-Vazquez and Quintana, 2018). Upon activation by tryptophan metabolites, AhR translocates into the cell nucleus and binds its partner, the AhR nuclear translocator (ARNT), to regulate expression of various immune response genes (Gutierrez-Vazquez and Quintana, 2018; Roager and Licht, 2018). These include genes involved in the suppression of hyper-inflammation, including Type I interferons, transforming growth factor β, and interleukin 10 (Figure 2A; Benson and Shepherd, 2011; Rothhammer et al., 2016; Yisireyili et al., 2017). Excess inflammation is one of the key factors contributing to pathogenesis of TB (Kaufmann and Dorhoi, 2013; Roca and Ramakrishnan, 2013). Thus, IPA could be explored as host-directed therapy to treat mycobacterial diseases (Kroesen et al., 2017; Palucci and Delogu, 2018). Importantly, activated AhR enhances the production of the interleukin 22, which stimulates the production of antimicrobial peptides and is also involved in inhibiting intra-macrophage growth of mycobacteria through increasing phagolysosomal fusion (Dhiman et al., 2009; Zelante et al., 2013; Memari et al., 2015; Shen and Chen, 2018). In addition, this cytokine stimulates the production of Th17 T cells, which produce interleukin 17, a protective cytokine against mycobacterial infection (Umemura et al., 2007; Scriba et al., 2008; Veldhoen et al., 2008; Shen and Chen, 2018). In line with this, double knock-out AhR^−/−^ mice are hyper-susceptible to *M. tuberculosis*, confirming the involvement of AhR in the control of mycobacterial infections (Moura-Alves et al., 2014). Recently, it has been shown that several anti-TB drugs bind to AhR, altering host defense mechanisms such as phagocytosis, and influencing the TB treatment response in a mouse model (Puyskens et al., 2019). These observations suggest that IPA may have potential in controlling mycobacterial disease by dampening excess inflammation and enhancing killing of mycobacteria. In the next section, we discuss three mycobacterial diseases, TB-meningitis, TB-HIV, and TB-diabetes for which the IPA treatment may be of relevance.

## IPA for TB-Meningitis?

TB-meningitis, an inflammation of meninges resulting from *M. tuberculosis* infection of the brain and spinal cord, mainly affects children and immuno-compromised patients (Wilkinson et al., 2017). It is a devastating disease with limited treatment options and unfavorable treatment outcomes (Chiang et al., 2014; Graham and Donald, 2014; Davis et al., 2018; Nguyen et al., 2019). Neurological damage is the most relevant sequelae of TB-meningitis (Thwaites et al., 2000; Rock et al., 2008; Chiang et al., 2014). Inflammation-mediated damage has been recognized as the major contributor to the irreversible neurological pathology as well as high mortality (Thwaites et al., 2004; Rock et al., 2008; Be et al., 2009; Thuong and Thwaites, 2017; Rohlwink et al., 2019). Hence, administration of anti-inflammatory drugs, usually corticosteroids, along with antimycobacterials is common practice (Thwaites et al., 2004; Prasad et al., 2016; Schutz et al., 2018). IPA is not only detectable in human serum but also in cerebrospinal fluid, showing its ability to cross the brain-blood barrier (Young et al., 1980). Interestingly, IPA has been shown to have neuroprotective effects (Chyan et al., 1999; Bendheim et al., 2002; Hwang et al., 2009) and is in early clinical development under the name of VP20629 for Friedreich’s ataxia, a neurodegenerative disease.^1^ Similarly, tryptophan metabolites prevented Aβ peptide-induced neurodegeneration *via* AhR activation (Platten et al., 2019; Maitre et al., 2020). An observational cohort study identified an association between low tryptophan concentration in cerebrospinal fluid and survival of TB-meningitis patients (van Laarhoven et al., 2018). Tryptophan is known to be catabolized by indoleamine 2,3-dioxygenase (IDO) upon inflammation (Kueck et al., 2018) as well as in response to tuberculosis infection (Gautam et al., 2018). The major product of this conversion, kynurenine, is an anti-inflammatory metabolite known to suppress central nervous system (CNS) inflammation *via* AhR activation (Cervenka et al., 2017; Crunkhorn, 2018; Figure 2B), consistent with the association of low tryptophan in the cerebrospinal fluid with improved survival (van Laarhoven et al., 2018). Likewise, it has been shown that IPA activates AhR and suppresses CNS inflammation (Rothhammer et al., 2016), and thus may also be beneficial in TB-meningitis, where mortality is driven by immunopathology (Graham and Donald, 2014; Davis et al., 2019).

High levels of the antioxidant glutathione have also been associated with the positive treatment outcomes among patients with TB-meningitis (Kalita et al., 2019). In this context, it is interesting to note that IPA is not only an antibiotic and an immune-modulator, but also a potent antioxidant that detoxifies reactive oxygen species, such as hydroxyl radicals (Karbownik et al., 2001, 2006; Hwang et al., 2009). IPA does not undergo autoxidation in the presence of transition metals making the molecule superior to most naturally occurring and synthetic antioxidants (Chyan et al., 1999). Thus, IPA could have additional positive effects on treatment outcome and irreversible neurological damage of TB-meningitis *via* its antioxidant properties (Wilkinson et al., 2017; Jain et al., 2018). Considering IPA’s brain pharmacokinetic, antimycobacterial, anti-inflammatory, and antioxidant properties, the molecule may find utility in the management of TB-meningitis (Wilkinson et al., 2017; Davis et al., 2018; Jain et al., 2018). Animal models are available to explore this therapeutic option (Tsenova et al., 2006; Tucker et al., 2016; Zhan et al., 2017).

## IPA for TB-Immune Reconstitution Inflammatory Syndrome?

TB-HIV is a dominant co-morbidity that accounted for 251,000 deaths in 2018 (WHO, 2019). Tuberculosis-associated immune reconstitution inflammatory syndrome (TB-IRIS) is an excessive inflammatory response among TB patients initiating anti-retroviral therapy (ART) leading to T-cell restoration (Lai et al., 2015). The condition is associated with increased proinflammatory cytokines, such as interferon γ and TNF-α (Lai et al., 2015; Walker et al., 2018). In a randomized control trial, the anti-inflammatory drug prednisone reduced the incidence of tuberculosis-associated IRIS (Meintjes et al., 2018). In a recent multi-cohort study, increased catabolism of tryptophan to kynurenine was associated with active TB compared to healthy subjects, and gradually reverted to baseline during the course of successful treatment (Collins et al., 2020). Increased kynurenine/tryptophan was associated with significant increases in IDO transcripts, suggesting that tryptophan catabolism is mediated by induction of IDO. The authors suggested IDO mediated tryptophan catabolism as a biomarker of TB disease progression as well as host-directed therapy (Collins et al., 2020). Accordingly, the early treatment with corticosteroids was effective in reducing the incidence of TB-IRIS and symptom severity (Sereti, 2020), partly due to increased IDO expression (Maneechotesuwan et al., 2008). Whereas the anti-inflammatory activity of IDO may hamper immune control of *M. tuberculosis* replication due to decreased proliferation of *M. tuberculosis* antigen-specific T cells (Mehra et al., 2013; Gautam et al., 2018), it may also be beneficial under conditions where mortality is driven by excessive inflammation, such as TB-IRIS. Since IPA phenocopies the anti-inflammatory effect of kynurenine *via* AhR (Rothhammer et al., 2016) and exhibits antimycobacterial properties, IPA could modulate the Trp-IDO-AhR axis to influence infection outcome to the benefit of the host in TB-IRIS management (Figure 2B).

## IPA for TB-Diabetes?

TB-diabetes co-morbidity is one of the major challenges in the management of TB, particularly considering the fast-growing incidence of diabetes in developing countries and the huge burden of TB (Dooley and Chaisson, 2009; Jiménez-Corona et al., 2013; Zheng et al., 2017). In a series of reports, IPA has been suggested to play a protective role against Type II diabetes, the major type of diabetes associated with TB (de Mello et al., 2017; Abildgaard et al., 2018; Tuomainen et al., 2018). The likelihood of developing diabetes has been shown to be reduced among individuals who have higher levels of IPA in their blood (de Mello et al., 2017). IPA has also been shown to reduce fasting glucose levels in rats (Abildgaard et al., 2018). Furthermore, a positive correlation of dietary fiber intake, insulin secretion, and IPA concentration in the blood was found, while IPA levels were negatively associated with inflammation markers (Kumar et al., 2013; Tuomainen et al., 2018). Consistent with these findings, the proportion of some of the IPA-producing gut bacteria is reduced among diabetes Type II patients (Larsen et al., 2010; Aw and Fukuda, 2018). Interestingly, a meta-analysis showed that the antihyperglycemic drug metformin, which also displays anti-inflammatory properties, has benefits in both prevention and treatment outcomes of TB patients with diabetes (Cameron et al., 2016; Zhang and He, 2020). Paradoxically, serum AhR ligand activity was higher in subjects with Type II diabetes, correlated with parameters of insulin resistance, and was a risk factor for diabetic nephropathy (Kim et al., 2013; Roh et al., 2015; Lee et al., 2020). The authors attributed AhR ligand activity to persistent organic pollutants such as tetrachlorodibenzo-para-dioxin, a dioxin with the highest known binding affinity to AhR (Mandal, 2005), although a causal relationship was not established. It has been proposed that activation of AhR by organic pollutants influences glucose tolerance *via* regulation of PPAR-α (Wang et al., 2011). Whether IPA-mediated AhR signaling influences glucose metabolism *via* the same pathways as environmental toxicants remain to be determined. Animal models are available to explore the antimycobacterial, anti-inflammatory, and anti-diabetic effects of IPA, and its potential to improve the clinical management of TB-diabetes (Martens et al., 2007; Srinivasan and Ramarao, 2007; Vallerskog et al., 2010).

## Conclusion and Outlook

The discovery that the gut microbiota metabolite IPA has antimycobacterial, anti-inflammatory, and antioxidant activity has a multitude of implications. In the context of drug discovery, this molecule presents an attractive chemical starting point for medicinal chemistry campaigns to generate more potent semi-synthetic IPA analogs, paving the way for the development of a novel broad spectrum antimycobacterial for the treatment of both TB and NTM lung disease. In the context of human microbiome and disease, IPA may present a functional link between the gut microbiota and host susceptibility to mycobacterial diseases. If confirmed, circulating IPA levels could turn out to be a useful prognostic marker and microbiota-based treatment strategies could be explored. Further analyses of IPAs immune modulatory and antioxidant effects in mycobacterial diseases may reveal adjunctive host-directed therapy opportunities, particularly in TB-meningitis and TB-diabetes patient populations. Lastly, the fact that this new antibiotic is a microbiota metabolite suggests that human microbiome-related metabolites may present an underappreciated source for the discovery of novel antibiotics (Donia and Fischbach, 2015; Milshteyn et al., 2018).

## Author Contributions

DN, MG, VD, and TD: writing – original draft preparation, review, and editing. TD: funding acquisition. All authors have read and agreed to the published version of the manuscript.

### Conflict of Interest

The authors declare that the research was conducted in the absence of any commercial or financial relationships that could be construed as a potential conflict of interest.

## Footnotes

^1^https://ClinicalTrials.gov/show/NCT01898884

## Abbreviations

IPA: Indole propionic acid
TB: Tuberculosis
NTM: Non-tuberculous mycobacteria
R03: Rule-of-3
AhR: Aryl hydrocarbon receptor
